# Vital Forces—The Closing Lecture of the Course on Chemistry, to the Students of the Savannah Medical College, Delivered March 10th, 1860

**Published:** 1860-06

**Authors:** N. A. Pratt


### SELECTIONS.

*Vital Forces—The Closing Lecture of the Course on Chemistry, to the Students of the Savannah Medical College, delivered March 10th*, 1860. By N. A. Pratt, M. D.

Gentlemen—It may appear strange that at this, the last hour of the course, I bring before you the subject of Force, to which the first lectures of the course were devoted. I have done so, however, intentionally, in order that, having followed me through the minutiæ of Forces, as exhibited in the inorganic and organic worlds, you may be the better able to take with me a general survey of the whole, and from a more eligible stand-point, study their relation to each other and to the world in and around us.

The ideas here recorded are such as have been suggested by reflection and study. Some of them are novel—and, on this account, are not so definite and clear as I would like, but I doubt not that future investigation will demonstrate that they point in the direction of truth. Without stopping to attempt to define *force*, you will remember that Gravity, Heat, Electricity, Magnetism, &c., are called *physical forces*, and have reference to *masses* of matter. Chemical forces are such as pertain to the *elements* and *molecules* of matter, and are subject to the laws of chemical affinity. The laws of both these different kinds of forces have been discovered, and are well understood; with them the physicist deals, and by the application of one or more of these laws, he can readily explain most of the pnenomena observed in the physical world.

With the physiologist, however, the case is different. In studying the origin, development and maintenance of organic structure, he finds himself involved in mystery and doubt; his physical forces, so far as his knowledge of them extends, are decidedly deficient; his laws of chemical affinity seem often to be contradicted and transcended, by intangible, unknown and incomprehensible agencies. Thus baffled in the phenomena of organic nature, by what he supposes a *subtle, invisible, uncontrollable power*—he calls it *organic* or *vital force*. To illustrate: A plant in full activity and growth draws from the air and soil its food, in the form of carbonic acid, ($CO, _2$) water, ($HO$,) and ammonia, ($N H, _3$) of these, with the assistance of sunlight, it constructs its full and frequently massive structure, with all the products of mature vegetable life; of the first two, woody fibre, (cellulose, lignin,) starch, gum, sugar, oils, &c.,—while for the production of the proteinacious or albuminous compounds, ammonia, in addition to these is required.

Now, this action of plant life is peculiar. By no other means can these stable compounds of the mineral kingdom ($CO_2$, $HO$ and $N H_3$,) be changed into the organic substances above mentioned. Heat, light, electricity—all the physical forces—even with the aid of chemical affinity, as at present understood, are powerless. This last, indeed, instead of lending a helping hand to the formation of these ternary and quaternary compounds, seems to exert all its energies in exactly the opposite direction. Chemical affinity, uncontrolled, is fully satisfied only when carbon and hydrogen have united with oxygen to form $CO$ and $HO$, and these will always be found through the processes of fermentation and putrefaction; they are the ultimate products of uncontrolled chemical affinity—they are in *the condition of most stable equilibrium.*

The same remarks apply with greater force to the phenomena of animal life. As the plant elevates *mineral matter*, binary compounds, one step of progressive development into a vitalized structure, made up of ternary or quarternary compounds, so the animal, taking as food the products of vegetable life, raises them a second grade higher, modifies the arrangement of the molecules, endows them with a more intense vitality, and places them on the higher platform of animal developement.

Thus, removed to a greater distance from the condition of most *stable equilibrium*, the elements C, H, N and O are united by a more precarious bond, and fall the more readily, under the chemical force, back again to the mineral kingdom, taking on the form of $CO_2$, $HO$, and $N H_3$.

That "principle," "agent," or "power," as it is called by different physiologists, which, acting through an organic cell, thus transcends known chemical affinities, and of the mineral kingdom constructs the organized fabrics of the vegetable and animal worlds, and stores them with their peculiar organic products, is what is called the "*vital force*," and to it your attention is invited.

This term, "vital force," you will at once perceive is simply *a name given to the unknown cause of a known effect.* We can investigate the laws of the *motions* and *equilibrium* of forces, but the history of their origin can never be written. All that we know of heat and electricity is a few of the laws of their action and their relation to each other. All that we may reasonably expect to discover of "the vital force," is no more than this—we hope no less.

We can have no sympathy with a once numerous class of physiologists, and, if we mistake not, with the larger body of intelligent physicians of the present time, who, observing the apparent antagonism of chemical towards "vital forces," believe that the former takes no part in the vital phenomena, but that they are controled entirely and arbitrarily by inherent organic agencies, separate and distinct from all known forces. Organic phenomena are not independent of the physical forces and chemical action; on the contrary, these all are necessary to organic existence, and "it has been proven by the most careful chemical investigation that organic bodies are constituted according to the same laws, and in their transformations follow the same laws that prevail in inorganic chemistry." Moreover, it is eminently unphilosophical to resort to imaginary forces to explain actual phenomena. In the study of nature, *natural* agencies and *known* forces should be exhausted before the *supernatural* are called into requisition.

Nor, on the other hand, can we agree with those who make physical and chemical forces alone account for *all* the phenomena of organic life, else we would be at a loss to account for the facts which daily meet our observation. The ovules of a plant standing side by side in the ovary, and in all respects subject to the same external conditions, exhibit remarkable differences in their subsequent career. Those which have been fertilized by the vitalizing action of the pollen, develope perfect seeds, while those prove abortive that, by accident or otherwise, have been deprived of such contact. The unfecundated egg, exposed to all the conditions of incubation, putrifies, forming a mass proverbial for its offensiveness, while that which has been impregnated by the mysterious contact of the spermatoza, under identical circumstances, developes a living automaton, with blood, muscles, viscera, heart, vessels, brain, nerves, organs of sense, endowed with sensation, consciousness, voluntary and involuntary movements. Here certainly we have something more than mere physical and chemical action—the dynamics are those of inorganic nature, the elements are all arranged in strict accordance with chemical law—but these are transcended and controlled by a *higher law*, to which, under the conditions of organic life, they are subject; this is evident. But, on the other hand, notice the peculiarities of this so-called "presiding essence," "principle," or "force,"—it is so dependent on external conditions, physical and chemical agents, that it is absolutely powerless without their assistance. An illustration will make this clear. A perfect seed, under suitable circumstances, according to a law impressed upon it by the Creator, will develop a plant altogether similar to the parent plant. If, however, the conditions of growth are not suitable, the germ remains dormant. Thus grain has been preserved for centuries in Egyptian monuments. So, also, a fecundated ovum or egg, carefully protected against all external forces, may be kept for a long time. The vital energies of the germ lie dormant, but not dead, still subject to that higher law of its nature, but powerless in the absence of that *force* which is to urge it forward to its destiny. The engineer is at his post, the engine, with its wonderfully perfect machinery, stands ready to obey the first impulse on its piston, but the motive power is still ungenerated.

This motive power, gentlemen, required by the germs of the seed and egg, can only be found in the physical and chemical forces to which they are exposed. Let the oxygen of the air have free access, under favorable conditions of heat and moisture, and carbonic acid will be formed, "vital force," no doubt, generated, and normal development ensue. Exclude the air, changing the conditions of heat and moisture, or not, as you please, no oxidation can go on, no "vital force" be generated, and no developement ensue.

We have thus, then, in the preceding discussion, shown that *what is generally called* "*vital or organic force*," as separate and distinct from physical and chemical force, *has no existence in nature.* We hope to make this plainer in the sequel.

After Liebig, the idea has extensively prevailed, and if I have not misconstrued the recorded opinions of most physiologists, the opinion is still prevalent, that amylaceous, saccharine and oleaginous food, after entering the blood, undergoes oxidation, and by its combustion, assisted by other chemical changes going on, generates the heat which is to keep up the normal warmth of the body, and at this favorable temperature the "vital force," by organizing the nitrogenous food, is able to keep life and activity in the animal. This being the general idea, it appears at once that this force must either be a very complex one, or else consists of several unknown forces grouped together under one name. An organic force must be present to digest food and change it to blood, another to remove exhausted tissue, another to replace it by new and living material—then there must be nervous forces, "excitor nerve force," to regulate motion, both voluntary and involuntary.* Each of these may be compound, and others may be necessary. Thus complicated is the common view of "vital forces," to which many cling with persistent tenacity.

Now the operations of Nature are eminently simple. Natural Science has shown that "perfect unity of plan amid infinite diversity of phenomena" is the principle on which the universe has been constructed. May we not look, then, for perfect unity amid apparent infinite diversity of force?

Refusing to accept, then, the common, and probably the most generally received opinion with regard to this force, can a better one be brought forward? Let us see. In the preceding discussion, we have made an analysis of it and found it to consist of two elements—

1st. A presiding "principle" or "agent," or, as for reasons given below, we prefer denominating it a *higher law*.

2d. A power or force which is necessary to put this law into execution, or to construct the organism in accordance with this law.

---

*Dr. Jackson. See Lehmann's Chemical Physiology, pp. 32–40.

These two elements give full expression to all the phenomena of the *organizing* force, the 2d to do the work required, the 1st to direct and control the 2d, so as to secure identity of form, power and properties, between parent and off-spring. "Nervous force," no doubt, is nearly related, if not part and parcel of the second, though we are unable, at present, to give it full expression, or trace its relations.

Reversing the order of the elements given above, we will discuss them in order. First, however, a few remarks on the relations of well known physical and chemical forces. It is a generally established principle among physicists of the present day, and will soon be one universally recognized, that physical and chemical forces are "correlative,"—that is, they are mutually convertible into each other. When heat has accumulated in a cannon ball until it has attained the temperature of about 900° F. it is able to produce undulations in the luminiferous ether, and light is produced, the ball is red hot. To eyes more sensitive than ours, the sensation of light may ensue at a lower temperature. *Heat* here has been *changed into light.* Light absorbed also produces the sensation of warmth—it is changed into heat. Electricity produces, or rather, is changed into magnetism, heat or light, as you have seen in a former lecture. Heat, also, and electricity, produce motion of masses, by transformation, and the reverse of this is also true—motion may be converted into heat and electricity. A nail hammered on an anvil, arrests the motion of the hammer. The force, as motion, is lost, but it becomes heat. Heat, produced by friction, is simply transformed motion. There is also an intimate relation between these and chemical affinity—they are also "correlative." Numerous instances might be given, but you will understand from the above what is the principle of "correlation" in modern philosophy. Exactly how these forces are connected, and by what laws they are related, has not yet been established for them all; but they are so intimately associated that one cannot exist except at the expense of another. Thus the relation of heat and motion has been ascertained, and a certain amount of one has been found to be an exact equivalent of the other. The general principle has been stated that whenever motion is arrested, heat is produced—the amount of heat is directly proportional to the quantity of motion lost. In a beautiful series of experiments by Joule, an English physicist, the following conclusions were deduced:

1st. *That the quantity of heat produced by the friction of bodies, whether solid or liquid, is always proportionnl to the force expended.*

2d. *That the quantity of heat capable of increasing the temperature of* 1 *lb. of water by* 1 *deg. F. requires for its evolution the expenditure of a mechanical force represented by the fall of* 772 *lbs. through* 1 *foot of space.**

The same results were obtained with mercury and cast iron, both of which were subjected to friction in a similar manner. Other experiments, by Kupffer,† on the expansion of metals, give nearly the same results.

This, then, is really the *mechanical equivalent of heat. There is no loss of force.* Mechanical force disappears, but it reappears as a definite amount of heat. As then there is certainly no recent and continual creation or destruction of *matter*, but the quantity remains the same from age to age, though existing in different forms and under different conditions, so certainly there is no recent and continual creation and destruction of *force;* the quantity remains the same from age to age. Though existing under different forms and conditions, they are constantly and continually changing into each other. Heat, light, electricity, chemical affinity, and mechanical force, constitute then one vast never-increasing, never-diminishing reservoir of energy, which, discharging itself through different channels, performs all the labor of the organic and inorganic worlds. They are, then, only different expressions or manifestations of the one universal and ubiquitous energy.

Now, cannot what are commonly called "vital forces" be reduced to the same category—are they not correlative with the forces above mentioned? We think so; and while in the present condition of Science, it is impossible to demonstrate the fact, there is no doubt that our inability is due to our ignorance, and we can only hope for the time when, with more perfect

---

*Phil. Trans., 1850, I. 61. Chem. Soc. Qu. J., III., 316.

†Phil. Mag., [4] XII. 393.

knowledge of life processes, and of the relations of physical and chemical forces, we may be enabled to elucidate the mysteries of organic life.

It is evident that in the development of the germ to the full organized structure, motion, separation and union of molecules, is necessary. What, then, is this force, which has been given above as the second compound of "organizing force?" Let an appeal to facts decide. There are two grades of organic life—the animal and vegetable. So, also, there are two grades of inorganic existence—the mineral and the elementary. In the union of elements to form the compounds of the mineral kingdom, a definite amount of force in the form of chemical affinity is called into requisition, and after union, that force exists as the bond which binds the elements together. Destroy this union, as in decomposition, and this chemical force is disengaged—it immediately, in the nascent state, *unites the elements into other forms*, if suitable conditions are present; if not, being indestructible, it can no longer exist as affinity, but quickly transformed, it appears immediately as one of its correlatives, heat, light, or electricity.

In the germination of seeds, warmth, moisture and oxygen are necessary. Entering the embryo, they secure the oxidation of a part of the starch and oily matter, and decomposition ensues—part of the carbon, as an ingredient, is broken from its union, its chemical affinity is set free, the remaining ingredients are transformed into sugar, gum, and other vegetable products.

The process of incubation is altogether similar—the same conditions are necessary. Oxygen is always absorbed, carbonic acid evolved, and the egg loses weight—the affinity disengaged, the organization of the blastema is changed, and blood, muscle, viscera, brain, nerves, &c., are formed. These simple, well-known facts of incipient organic development, have been variously interpreted. The common opinion is, that the presence of heat, moisture and air are the conditions of growth; they are the "*vital stimuli*" which arouse the dormant or latent "*germ force*" into action. Now this so called "germ force" *is and can only be a fiction.* The convenient hypothesis of latent forces must give way to the logical doctrine of "correlation," "for it is now coming to be generally acknowledged that all force (from its very nature) must be *active* in some mode or other; that force can neither originate *de novo*, nor cease to be operative under some form; and that in every case in which force *seems* to be annihilated, it merely changes its "*modus operandi.*"* "Vital force" must then be some other force *ab externo*, either acting under modified circumstances, or else entirely transformed. The physiologist, Carpenter, supposes that, in the case of growing plants, *light* is the physical force which, acting through the "organic substratum" in the vegetable cell, is transformed into vital force, and in germination, incubation, and growth of animals, heat is the physical force thus transformed,* and it, of course, follows, that chemical force simply arranges the molecules under the control of the vital forces. More recently, the theory has been advanced and ably supported by Dr. Jos. LeConte, of Columbia, S. C., that *chemical force* is that which is immediately transformed into the "vital force." According to this theory, "the conversion of *physical* into *vital* force seems impossible, without passing through the intermediate condition of *chemical force.*"† *Vital* force is then transformed *chemical* force. Many facts can be consistently explained on this theory which are inexplicable on the other, e. g., in the seeds of all plants is laid up a store of nutritious matter which supports the germinating plantlet until its roots and leaves are sufficiently developed to support themselves; this nutritious matter, as also that on which pale plants feed, is more highly carbonized than the vegetable products formed—the excess of carbon is consumed by the oxygen to which the seed must always be exposed—in its change, affinity is disengaged and is transformed into a force which organizes the remainder. So, also, in incubation and in the respiration of animals, oxidation always takes place, and is the immedtate source of the vital force.

We know too little of the relations of heat and chemical action, as correlatives, to decide which of these forces has the precedure in the order of Nature—probability most certainly leans

---

*Carpenter's Human Physiology, 6th Am. Ed. pp. 141-149.

†See an admirable paper on "*The correlation of Physical, Chemical and Vital Force, and the Conservation of Force in Vital Phenomena,*" read before the Am. Association for the advancement of Science, Aug. 1859, by Joseph LeConte, M. D., Professor of Geology and Chemistry in South Carolina College, Columbia.

in favor of the latter. But why acknowledge, as both these theorists have done,* the existence of a *vital force* into which heat, light, or chemical force is transformed? Why must the well-known force be changed into a suppositious, mythical force? Because our knowledge of chemical affinity is too limited to explain many of the phenomena of organic life, shall we grasp at the shadow for the substance, and rest satisfied by attributing the phenomena to a "*vital force?*" There is no more proof of the existence of a force of this kind than there is of the *Catalytic* force or presence action of Berzelius and Mitscherlich. This latter doctrine is by all considered only a convenient fiction, bringing together under one arm a number of decompositions not provided for in the theory of chemical affinity, as at present understood, but which will be fully explained by new investigations.† If "vital foree" is a convenient term, used in similar manner, no objections can be made. Very few, however, look upon it in this light.

The results of the union and disunion of chemical bodies are greatly dependent on the circumstances under which chemical action takes place, a few instances may be mentioned. Sugar in the blood, or elsewhere, under the action of an excess of oxygen, will give rise to *carbonic acid* and water, with less oxygen—*oxalic acid* and water is formed, if, in contact with animal matter in incipient putrefaction, *alcohol* and carbonic acid is formed —if the putrefaction has advanced somewhat, or if the temperature is raised, *mannite* or *lactic* acid or *butyric acid* is formed. Alcohol, in different proportions, with sulphuric acid, at different temperatures, is changed into ether, ($C_4 H_5 O$,) or olefiant gas, ($C_4 H_4$ ), or not at all. Now all of these are products of undoubted chemical action, though modified by the external conditions to which it is exposed. Modern investigation into the nature of complex organic compounds, confirm more and more the belief that they are all compounded of more simple proximate elements, one of which the "*adjunct*" unites with the other, whether acids or bases, without destroying their characteristic properties, thus forming what are called the "*conjugate*"

*"On the Mutual Relations of the Vital and Physical Forces." Phil. Mag., 1850.

*Graham's Elements. 2d Am. Ed.

acids and bases. May not these "compound elements," the union of which is not well understood, be the "elements" of organic chemistry, and subject to laws which are yet to be discovered? May not, then, chemical force as such, under the conditions to which it is exposed in the animal body, be considered amply adequate to the production of all organized structure? Future investigation only can answer. Is not this, however, a more philosophic view than that which supposes a special organic force?

Having thus shown that physical and chemical forces must and can only be the active motive power in the phenomena of organic life, what is that first component of the organizing force, that "higher law" which controls and directs in organizing? This presiding "agent," "principle," or "essence," or germ force, as it has been called, is not a *force.* All force exerted by the plant or animal is drawn *ab externo*—as shown above, no part whatever can exist as an inherent property—the germ cell (germinal vesicle) can only develop under conditions which produce chemical action. This action is the vital force, but there is a law which in every case supervises the development of the germ. The acorn must become the oak, and not the pine—the spore of the fern must reproduce its parent, and not the mushroom—the ovum of the monkey ita own, and not the human kind. This law, *to reproduce itself* under the requisite material conditions, was implanted in the germ cell by the hand of creative power, and every successive cell is subject to the same. Let it be remembered here that the term law, as here used, implies no *coercive energy* in itself, like other laws of nature, it must be understood to mean, *only the human expression of the plan on which Divine power seems to operate.*

The study, then, of all phenomena, organic and inorganic, leads us back through paths constantly converging, and all ultimately meeting at the one Great Author of All; and here we must stop. What, then, are the laws of heat, light, electricity, gravity, chemical affinity? Simply different manifestations of the one *Ubiquitous and Omnipotent Will.*

What are "vital forces?" Simply the action of physical and chemical force in developing the germ and maintaining the life of the organism, in accordance with a plan or law devised by the Almighty Creator of all.—*Savannah Journal of Medicine.*

